# Defect processes in F and Cl doped anatase TiO_2_

**DOI:** 10.1038/s41598-019-55518-8

**Published:** 2019-12-27

**Authors:** Petros-Panagis Filippatos, Nikolaos Kelaidis, Maria Vasilopoulou, Dimitris Davazoglou, Nektarios N. Lathiotakis, Alexander Chroneos

**Affiliations:** 1 Institute of Nanoscience and Nanotechnology (INN), National Center for Scientific Research Demokritos, 15310 AgiaParaskevi Athens, Greece; 2 Faculty of Engineering, Environment and Computing, Coventry University, Priory Street, Coventry, CV1 5FB United Kingdom; 3 Theoretical and Physical Chemistry Institute, National Hellenic Research Foundation, Vass. Constantinou 48, GR-11635 Athens, Greece; 4 Department of Materials, Imperial College, London, SW7 2AZ United Kingdom

**Keywords:** Chemistry, Materials science

## Abstract

Titanium dioxide represents one of the most widely studied transition metal oxides due to its high chemical stability, non-toxicity, abundance, electron transport capability in many classes of optoelectronic devices and excellent photocatalytic properties. Nevertheless, the wide bang gap of pristine oxide reduces its electron transport ability and photocatalytic activity. Doping with halides and other elements has been proven an efficient defect engineering strategy in order to reduce the band gap and maximize the photocatalytic activity. In the present study, we apply Density Functional Theory to investigate the influence of fluorine and chlorine doping on the electronic properties of TiO_2_. Furthermore, we present a complete investigation of spin polarized density functional theory of the (001) surface doped with F and Cl in order to elaborate changes in the electronic structure and compare them with the bulk TiO_2_.

## Introduction

Transition metal oxides such as titanium dioxide (TiO_2_) are considered as an extremely important class of materials due to their intense catalytic activity, superior chemical stability, long life cycle and high electrical conductivity^1–10^. Particularly, anatase TiO_2_ has been extensively studied because of its abundance and non-toxicity, high photocatalytic activity and responseto visible light arising from high its absorption coefficient and reflectivity^1–10^. Moreover, it represents the work-horse photoanode material in dye-sensitized solar cells (DSSCs)^11–16^, whereas recently has been widely applied as the device electron transport material in both organic-inorganic halide perovskite (PSC) and organic solar (OSC) cells^17–25^. Nevertheless, TiO_2_ exhibits a wide band gap of around 3.2 eV^26,27^ that limits its absorption in the visible and, especially, in the near infrared (NIR) region^7^. A common way to reduce the band gap of TiO_2_ is through doping with appropriate elements which has also significant impact on its electronic structure. A vast variety of literature reports previously demonstrated that by doping TiO_2_ with nitrogen (N), halogens such as fluorine (F) and chlorine (Cl), or several transition metal ions such as zinc (Zn) or nickel (Ni), significant changes in its electronic structure are observed^28–34^. Particularly, the formation of mid gap states resulting in a band gap reduction was evident. For instance, when titanium dioxide is doped with Ni the band gap decreases to 2.57 eV,^33^ whereas when it is doped with Cl mid gap states are formed and the band gap decreases to 3 eV^31^. Progress on the photocatalytic performance and heterogeneous catalysis of TiO_2_ and other oxides such as ZnO has also been accomplished by the introduction of oxygen vacancies^35,36^ combined with metallic doping.

Focusing on other defect related projects regarding the TiO_2_ and its application to photocatalysis, it is seen that TiO_2_ has a fast recombination of the conduction band electrons and valence band holes and as a result it is not a satisfactory photocatalytic for organic degradation. In order to solve that problem as well as to reduce the large band gap, it is seen in the literature that many doped models of TiO_2_ can have improved photocatalysis. For example, N doped TiO_2_^37^ and Nb doped TiO_2_^38^ is reported to have better photocatalytic properties than pure TiO_2_.

Although a profound band gap reduction can be beneficial to the material’s photocatalytic activity as it results in higher absorption of visible light, it might create mid gap states that usually act as charge traps hence having a negative impact on the performance of organic and perovskite solar cells utilizing TiO_2_ exclusively as electron transport/extraction material^39^. In those cases the photocatalytic ability of TiO_2_ should be suppressed as it degrades its interface with organic/perovskite semiconductor. The formation of mid gap states upon doping of TiO_2_ is therefore undesired in OSCs and PSCs. In the present study, fluorine and chlorine doping of the bulk and surface TiO_2_ is studied via Density Functional Theory (DFT) in order to examine the electrical structure before and after doping with F and Cl and investigate the potential improvement in the photocatalytic activity of the TiO_2_. Moreover we investigated many different defect sites for the F and Cl in the bulk system and we also calculated for the first time the interstitial sites and the changes in electrical properties of the (001) TiO_2_ surface after the F and Cl doping. Total density of states (DOS) and partial DOS (PDOS) of the energetically minimum sites of the defects are considered in order for the electrical structure changes to be fully understood. A band gap reduction is evident in both cases. Moreover, the formation of mid gap states in all cases is also predicted. Such states are highly beneficial for the photocatalytic applications of TiO_2_ though they can be detrimental for OSCs and PSCs performance as they constitute trap sites for the photogenerated charge carriers thus significantly reducing the device photocurrent.

## Results and Discussion

### Bulk anatase TiO_2_

**Figure 1:**
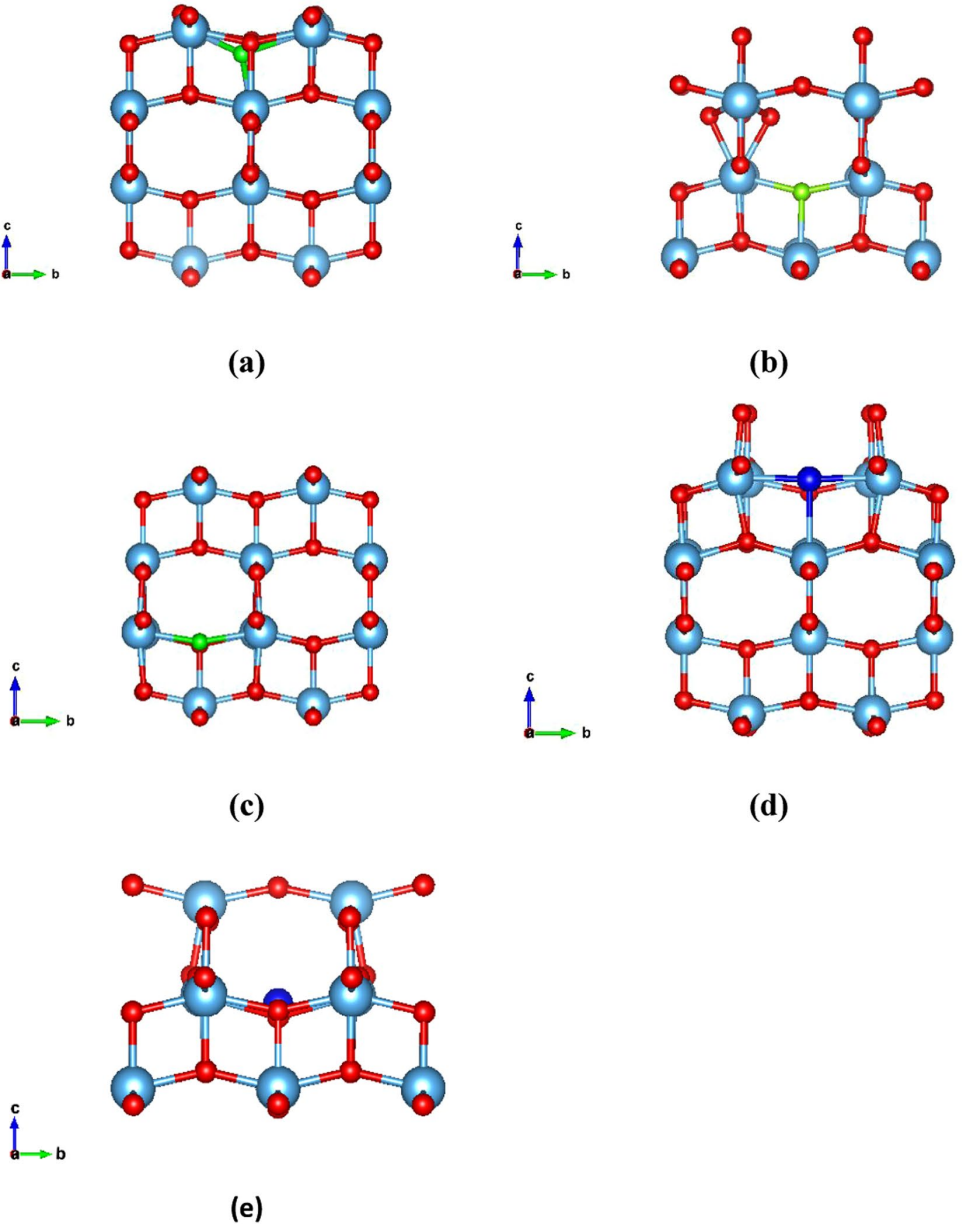
(**a**) The minimum energy structure of fluorine interstitial doped anatase TiO_2_, (**b**) The minimum energy structure of the F doped bulk TiO_2_ where F occupies an oxygen site, (**c**) The minimum energy structure of the substitutional F on the bulk anatase TiO_2_, (**d**) The minimum energy structure of the chlorine substitutional doped bulkanatase TiO_2_, (**e**) The minimum energy structure of the chlorine interstitial doped bulk anatase TiO_2_.

**Figure 2:**
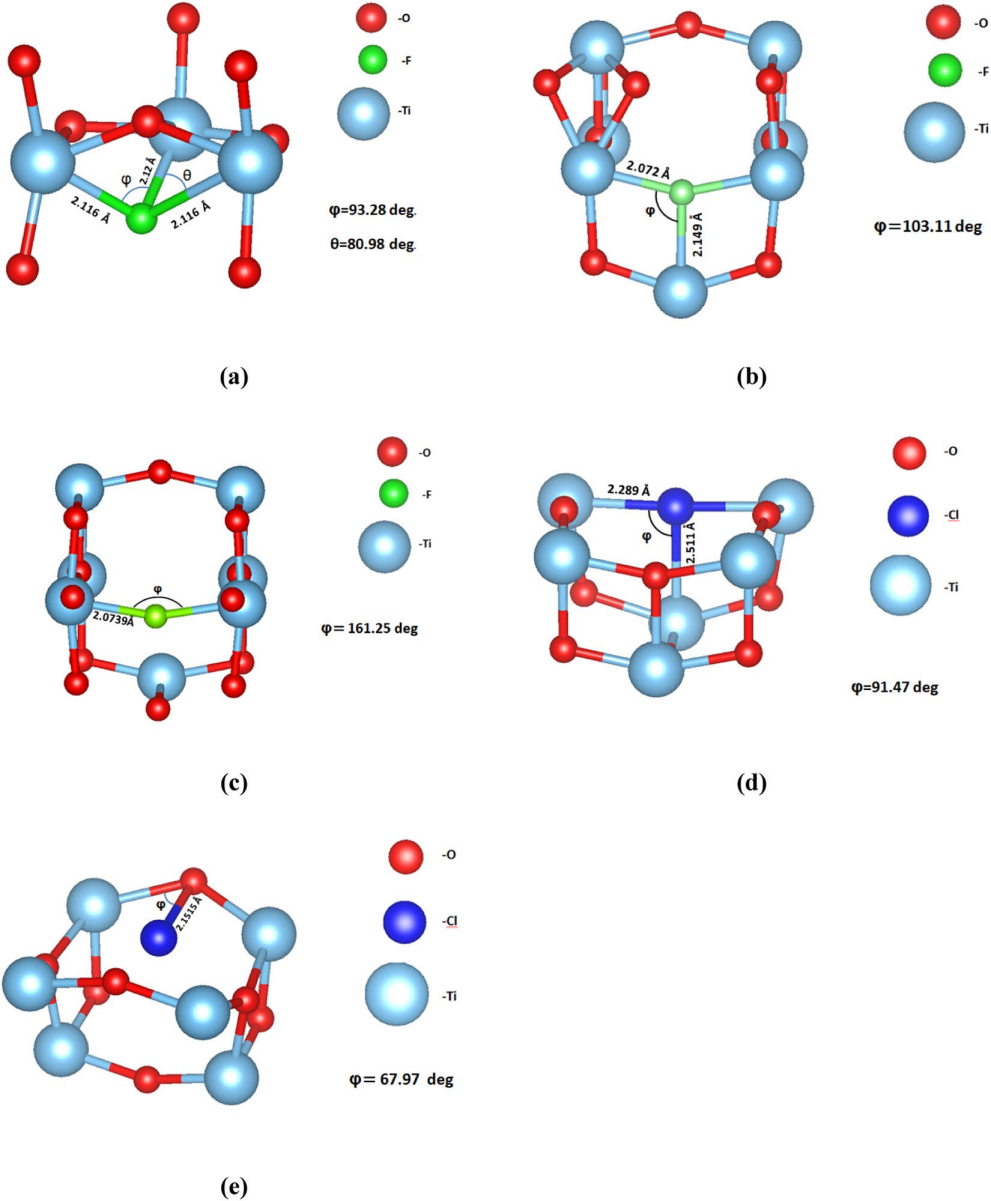
(**a**) The fluorine interstitial in the bulk TiO_2_, (**b**) The fluorine interstitial in the bulk TiO_2_ when the oxygen atom is displaced, (**c**) The fluorine substitutional in the bulk TiO_2_, (**d**) The Chlorine substitutional in the bulk TiO_2_, (**e**) The Chlorine interstitial in the bulk TiO_2_.

There are three polymorphs of TiO_2_ (rutile, anatase and brookite) with the anatase being the prevalent choice for photovoltaic applications as it has superior photocatalytic properties^40^. The crystal structure for the anatase is tetragonal with space group I4/amd and its experimental structural parameters calculated from neutron diffraction are a = 3.782 Å, b = 3.782 Å and c = 9.502 Å^41^. Our theoretically calculated lattice parameters of the anatase are a = 3.804 Å, b = 3.804 Å and c = 9.729 Å which also agree with other theoretical results^42,43^. The percentage of our dopants was 1 dopant atom per 109 atoms of TiO_2_ (0.91% doping). In the surface system we calculated 1 dopant atom per 96 atoms of TiO_2_ (1.04% additional doping). As regards the density of F and Cl atoms in the bulk systems, we calculated that for the F interstitial we have a density of 7.863 10^20^ cm^−3^, for the F substitutional we have a density of 7.872∙10^20^ cm^−3^ and lastly for the Cl interstitial and Cl substitutional we have a density of 7.825∙10^20^ cm^−3^ and 7.829 10^20^ cm^−3^ respectively. On the other hand, for the surfaces, we calculated a density of 4.451∙10^20^ cm^−3^ for the F interstitial and 4.423∙10^20^ cm^−3^ for the Cl interstitial. According to our DFT calculations, the F atom is stable either as an oxygen substitutional defect or an interstitial defect in the bulk TiO_2_ system. We found that in the case of interstitial, the fluorine atom is located at a distance of 1.983 Å from the nearest oxygen atom (see Figs. 1a and 2a) in agreement with previous studies^33^. Examining thoroughly the various defect formations, we concluded that the minimum energy configuration is when fluorine occupies an oxygen site displacing the oxygen to an interstitial site, as seen in Figs. 1b and 2b. Moreover, the simple substitution of an O atom with F has also been examined (Figs. 1c, 2c**)**. Concerning Cl atom doping of bulk TiO_2_, we show that the Cl can either substitute an oxygen atom or relax in a substitutional position, at a distance of 2.15 Å from the nearest oxygen atom (Figs. 1d,e and 2d,e).

**Figure 3:**
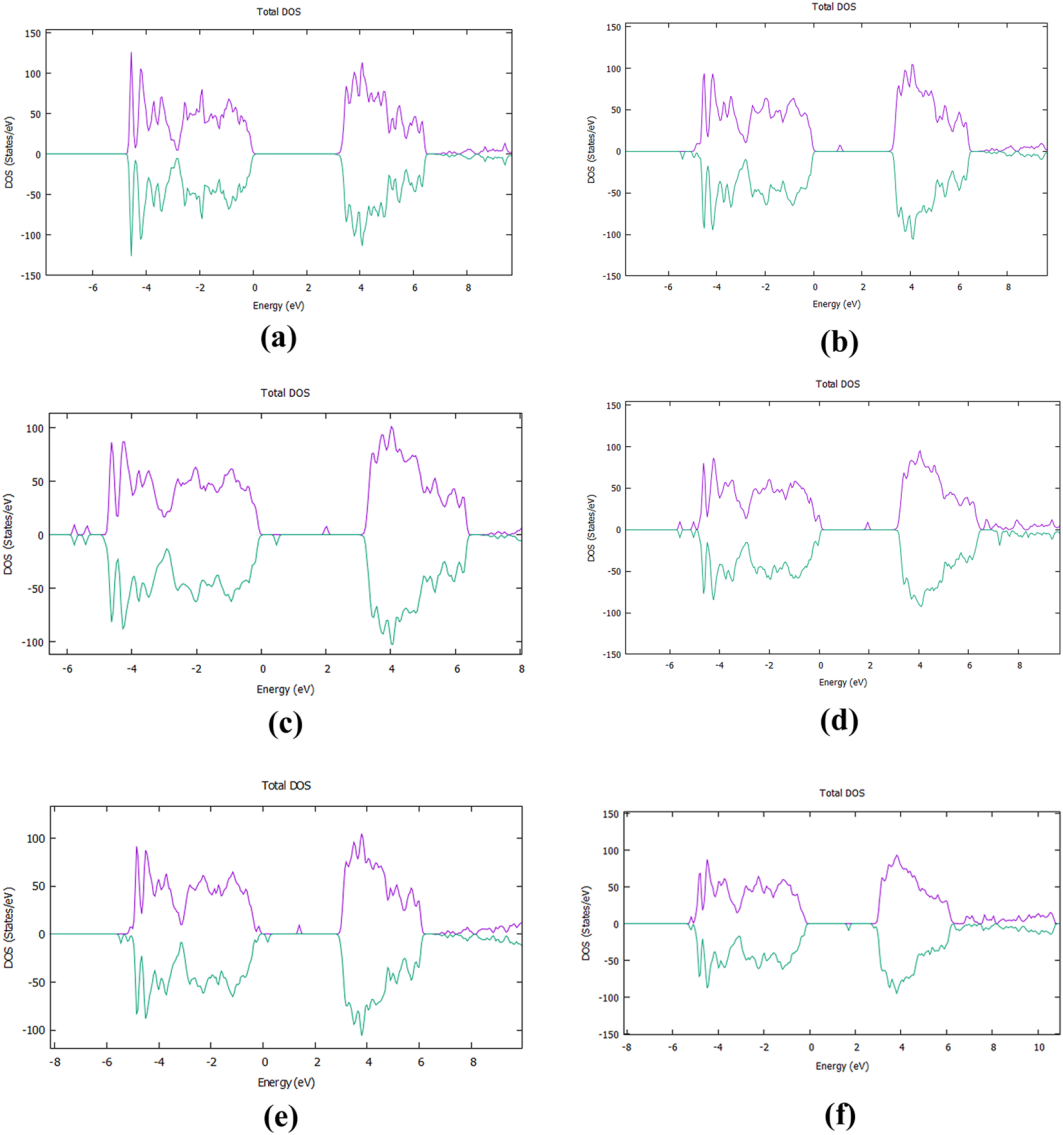
(**a**) DOS of the undoped bulk anatase TiO_2_, (**b**) DOS of interstitially F - doped TiO_2_ (**c**) DOS of F:TiO_2_ with F substituting an Oxygen site and displacing it to an interstitial site. (**d**) DOS of the F:TiO_2_ when F is a substitution to O, (**e**) The Density of States graph of the Cl-Doped TiO_2_ with Cl as an interstitial, (**f**) The Density of States graph of the Cl:TiO_2_ when Cl is a substitution to O.

For each supercell, we calculated the DOS (Fig. 3(a)–(f)) and, in Fig. 3a, the total DOS of the pure TiO_2_ is shown as a reference. Our calculations were performed with DFT + U model with the Hubbard-U parameter equal to 8.2 eV^33,34^. We calculated the band gap at 3.14 eV, in agreement with previous theoretical studies^33,34,44^, which show a band gap narrowing from 3 to 3.16 eV and close to the experimental value of 3.2 eV.

**Figure 4:**
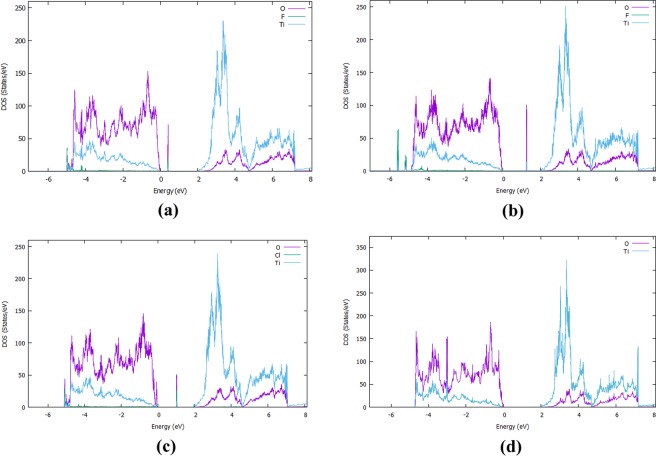
(**a**) The Projected DOS (PDOS) of the bulk F:TiO_2_ with F an interstitial, (**b**) The Projected DOS of the bulk F:TiO_2_ with F occupying an oxygen site and displaying the oxygen to an interstitial site, (**c**) The Projected DOS of the Bulk Cl:TiO_2_ with Cl an interstitial, (**d**) The PDOS of TiO_2_.

**Figure 5:**
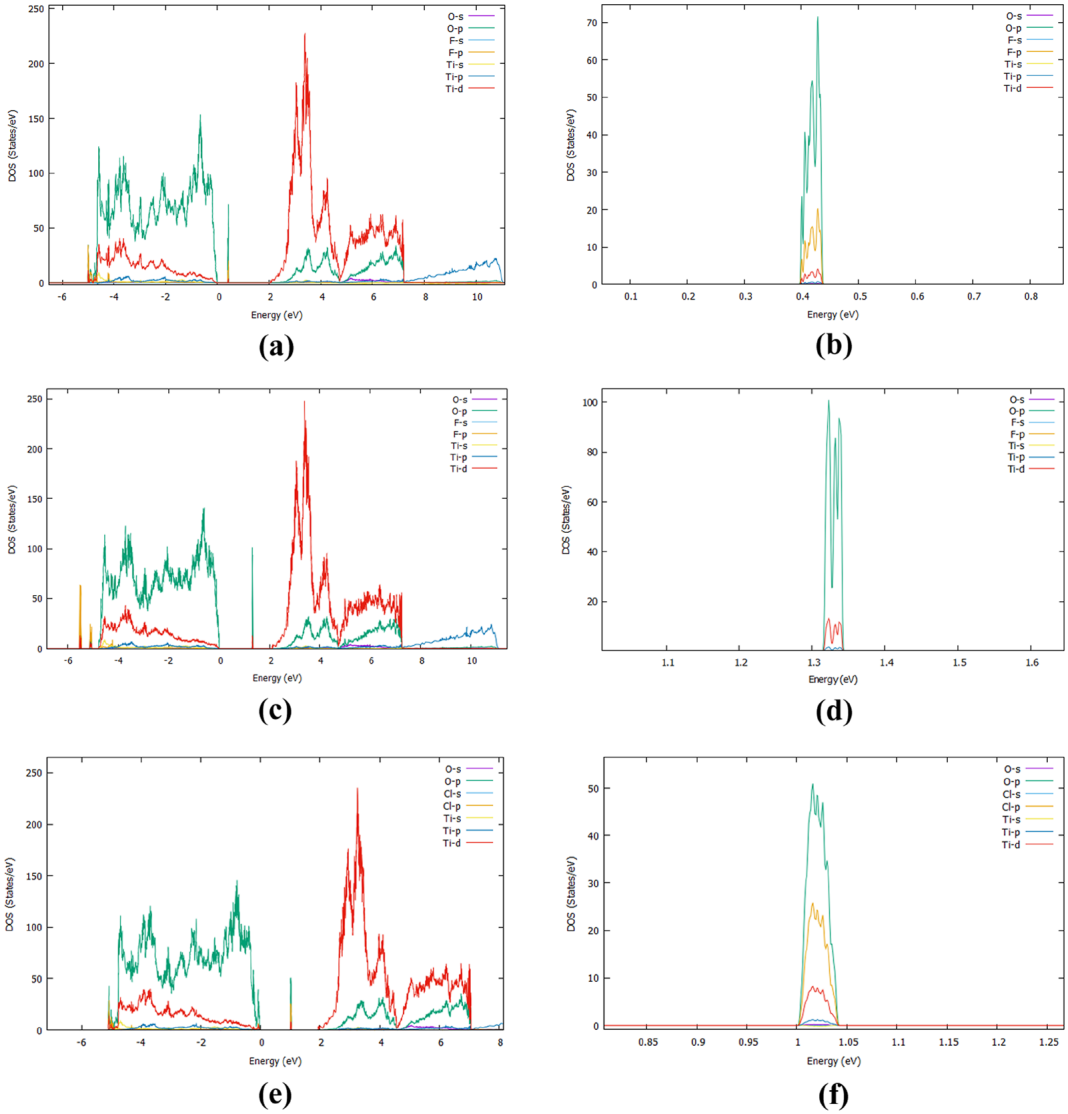
(**a**) The PDOS coming from the orbitals for the bulk F:TiO_2_, (**b**) The orbitals contribution to the mid gap rise of the bulk F:TiO_2_, (**c**) The PDOS from the orbitals of F:TiO_2_ when F occupies an oxygen position and displaces the oxygen to an interstitial site, (**d**) the orbitals contribution to the mid gap rise of F:TiO_2_ when F occupies an oxygen site and displaces oxygen to an interstitial, (**e**) The PDOS coming from the orbitals for the bulk Cl:TiO_2_, (**f**) The orbitals contribution to the mid gap rise of the bulk Cl:TiO_2_.

As we show in Fig. 3b, the F interstitial in the bulk TiO_2_ gives rise to a small peak inside the band gap at approximately 0.98 eV above the valence band (VB) and it also decreases the band gap to 3.04 eV. When an oxygen atom is replaced by a fluorine, the mid-gap impurity band is shifted towards the conduction band (Fig. 3d). Figure 4(a)–(d) represents the PDOS of doped and undoped bulk TiO_2_. In order to fully understand the emergence of the mid-gap peak in the case of the F interstitial, we calculated the partial DOS which is shown in Fig. 4a. Figure 5(a)–(f) examines in more detail the PDOS and the orbital contribution to the mid gap rise. There, one can see that the conduction band is mainly attributed to Ti while the valence band to O. In more detail,in Fig. 5a, it is shown that the main contributions to the valence and the conduction bands are attributed mainly to the O-2p and Ti-3d respectively. As far as the rise of mid-gap states (Fig. 5b) is concerned, we found that it is mainly attributed to the O-2p and F-2p orbitals. Concerning the minimum energy configuration with a fluorine replacing an oxygen which is displaced to an interstitial, two mid-gap levels emerge, one at 0.48 eV and one at 2.02 eV above the VB maximum. Moreover, it is seen that the band gap decreases by approximately 0.10 eV, reaching a value of 3.04 eV. To shed more light on these mid-gap peaks, a PDOS calculation was conducted again and presented in Fig. 4b. As one can see in Fig. 5d, the main contributions to these mid-gap levels come from O-2p and Ti-3d with and only a minimal contribution originates from fluorine, unlike the previous case of the F interstitial (Fig. 5b). A similar DFT research was conducted by Valentin and Pacchioni^45^ and they calculated that when F is inserted as a substitutional defect, the band gap is reduced to 3.08 eV which is also in a good agreement with the present results. Samsudin and Hamid^46^ have done an important experimental work on the band gap engineering of anion doped TiO_2_ and they calculated that the band gap of TiO_2_ is significantly decreased to 3.02 eV, which is again consistent with the present DFT work.

For the bulk Cl:TiO_2_, in the case of a Cl interstitial, the DOS (Fig. 3d) shows peaks at 0.19 eV and 1.38 eV above the conduction band minimum and, in addition, the band gap of the bulk TiO_2_ is significantly reduced to 2.89 eV. Looking at the PDOS of the bulk with Cl interstitial, we see that the valence and the conduction bands mainly consist of Ti-3d and O-2p and the mid-gap peak of O-2p and Cl-2p states. For the case of Cl substituting an O, one can see that a mid-gap peak appears again, at 1.77 eV, while the band gap is reduced to 2.84 eV (Figs. 1d and 2f). Focusing on the experimental work of Sun *et al*.^47^ it is seen that the band gap of the Cl:TiO_2_ reaches a value of 2.98 eV. This experimental work is also in agreement with our calculations, therefore it is suggested that band gap engineering of TiO_2_ can be used to a number of applications such as photocatalysis.

### Surface of anatase TiO_2_

**Figure 6:**
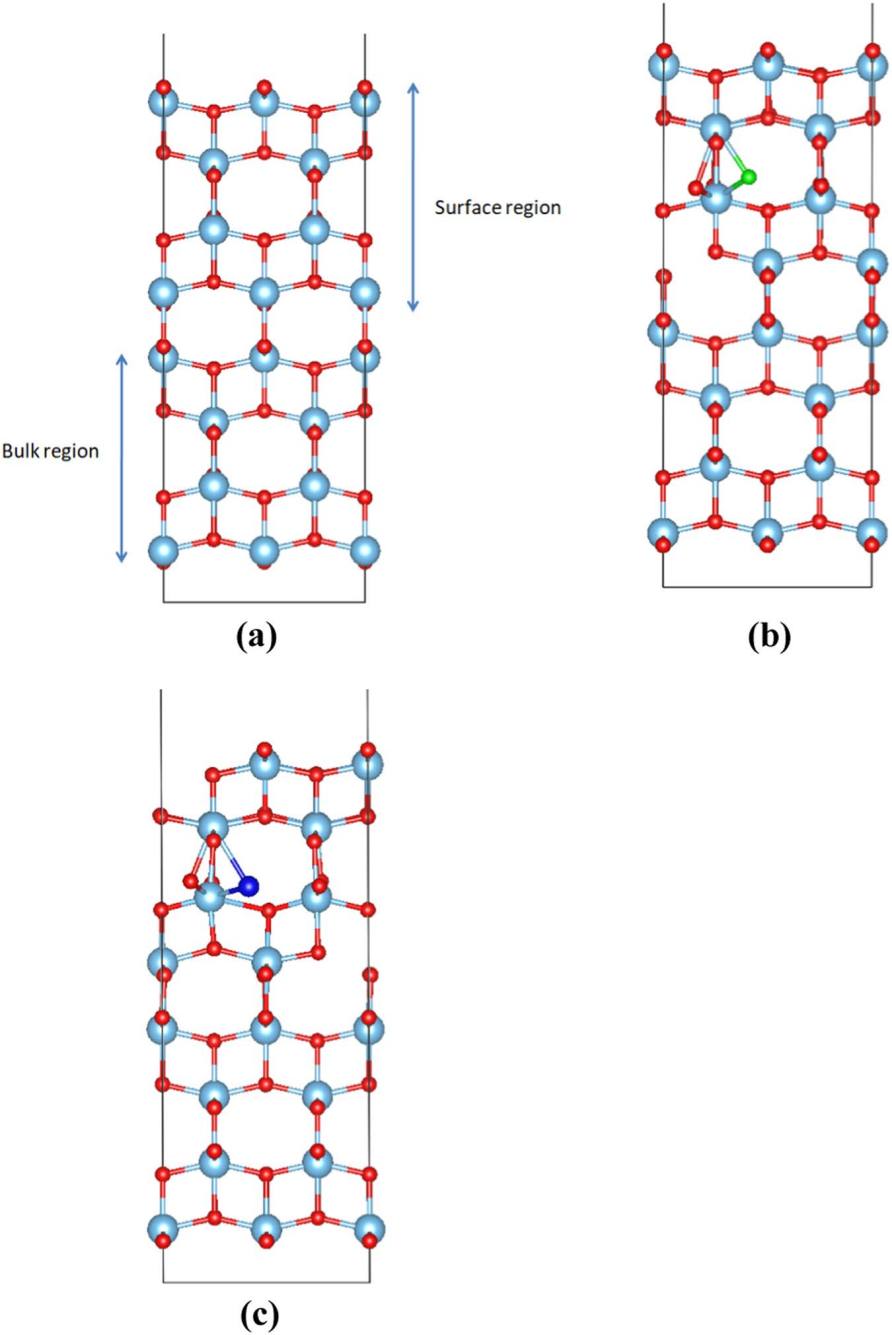
(**a**) The model for the TiO_2_ (001) surface where the bottom 4 layers represent the bulk region and the top 4 layers the surface region, (**b**) The F doped TiO_2_ (001) surface, (**c**) The Cl doped TiO_2_ (001) surface.

In order to develop better photocatalytic materials with visible light response and high activity, such as TiO_2_, more attention should be paid to surface doping with atoms or molecules. However, theoretical investigations of the effect of the surface doping on the surface electronic structure of TiO_2_ are at present scarce compared to the studies concerning the bulk system^48^. The aim of this section is to investigate the structural and electronic properties of the F- and Cl- doped TiO_2_ surface by using a DFT + U, in spin polarized calculations. We calculated the interatomic distances and angles, the electronic density of states of the undoped and doped TiO_2_ surface, as well as the changes of the band gap when the system is doped. For the simulation of the surfacewe used a slab model with a vacuum of 14 Å thickness vertical to the (001) plane and with periodic boundary conditions in the other directions. In our surface system, the top 4 layers were fully relaxed while the bottom 4 layers were kept fixed in order to simulate the bulk area. We chose this particular surface because it is a common choice in other studies concerning the absorption of different atoms and molecules, like CO_2_^49^, but the interstitial-doping with fluorine or chlorine atoms has never been studied before. Instead, the (101) surface has mostly been considered for fluorine and chlorine doping^50^. However, the (001) TiO_2_ surface is considered one of the most highly energetic surfaces of the TiO_2_ and, as a result, it often plays the role of the active site in photocatalytic reactions^51–55^. Τo the best of our knowledge only Zhou p. *et al*.^56^ have studied the TiO_2_ (001) surface in the case of F doping as a substitution of an O atom. The undoped configuration is shown in Fig. 6a while the minimum energy structures for F and Cl doped TiO_2_ (001) surfaces are shown in Fig. 6b,c respectively.

**Figure 7:**
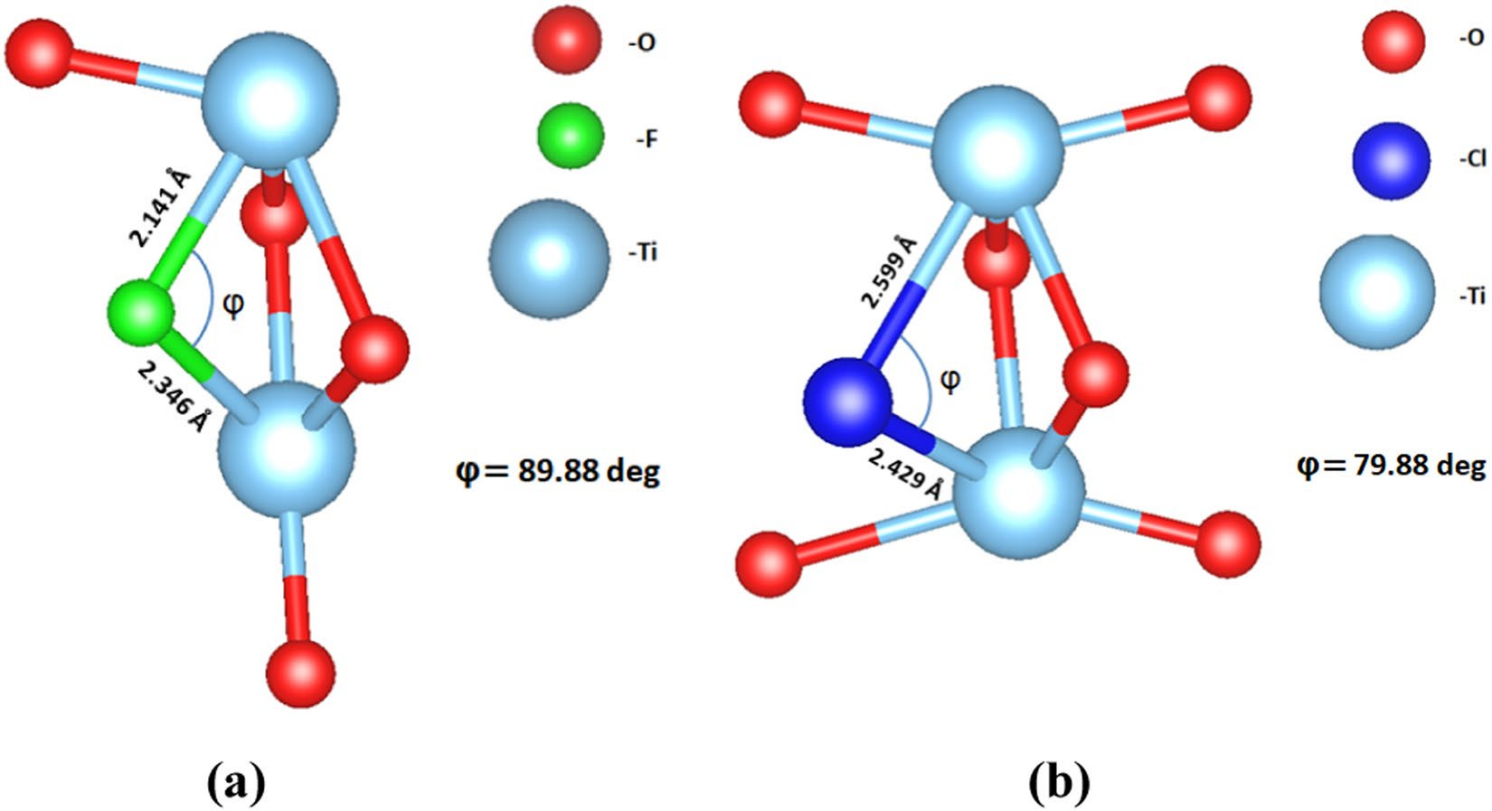
(**a)** Fluorine interstitial in the (001) TiO_2_ surface, **(b)** Chlorine interstitial in the (001) TiO_2_ surface.

**Figure 8:**
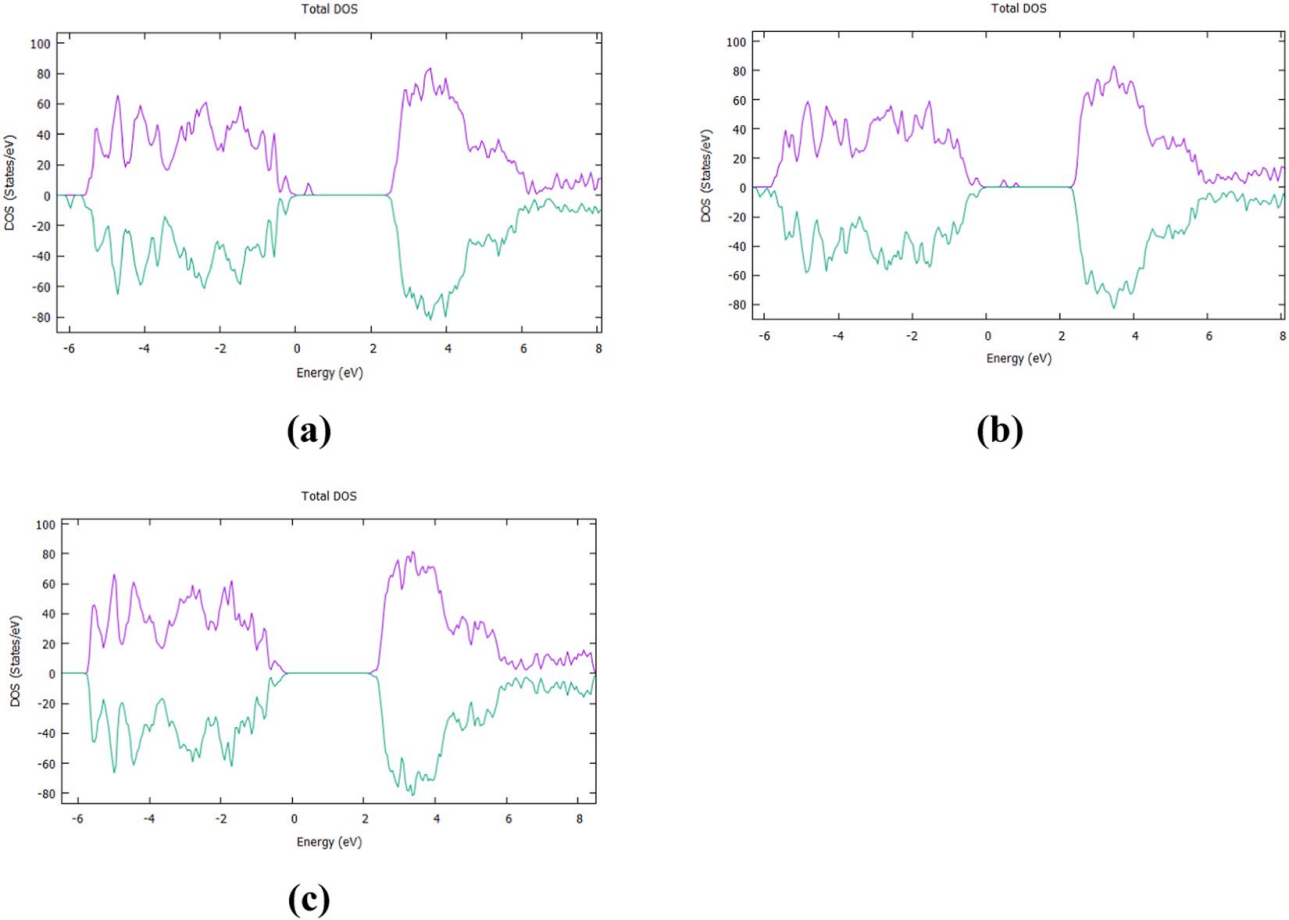
(**a**) DOS of F-doped TiO_2_ (001) surface with F an interstitial, (**b**) DOS of Cl:TiO_2_ (001) surface when Cl is an interstitial. (**c**) DOS of (001) surface of anatase TiO_2_.

According to our DFT calculations, when fluorine is inserted in the (001) surface as an interstitial (Fig. 7a), it is located at a distance of 2.00 Å from the nearest oxygen while Cl (Fig. 7b) at 2.18 Å. Looking at the DOS for each supercell, in Fig. 8a it is seen that the F interstitial at the surface produces a small impurity band peak at approximately 0.35 eV above the VB. On the other hand, as it is seen in Fig. 8b, when the surface is doped with a Cl atom, two mid-gap peaks arise, which are observed at approx. 0.55 eV and 0.93 eV above the valence band. Concerning the band gap, when the TiO_2_ surface is doped with F, the band gap is predicted at a value of 2.24 eV whereas for the Cl–doped surface, the calculated band gap reaches a value of 2.31 eV. The band gap of the undoped TiO_2_ surface calculated at 2.37 eV(Fig. 8c) in agreement with other theoretical studies concerning (001) TiO_2_ surfaces^57^. Therefore, the insertion of F or Cl on the (001) surface, has a minor effect on the band gap of the TiO_2_ system.

## Conclusions

DFT calculations were performed for fluorine and chlorine-doped anatase TiO_2_ bulk and surface structures in order to evaluate the effect this kind of doping on the band gap and the electronic structure of TiO_2_. Both substitutional and interstitial halogen defects were predicted. In all cases, occupied mid-gap states attributed to a hybridization of O-2p with halogen 1 s orbitals were also predicted. Although such states are beneficial for the oxide’s photocatalytic activity as they significantly reduce the optical band gap with respect to that of undoped TiO_2_, they might be detrimental for its application as electron transport material in other classes of photovoltaic devices such as organic and perovskite solar cells.

## Methods

### Computational methodology

We performed periodic DFT calculations using the CASTEP program^58,59^. The Perdew, Burke and Ernzerhof (PBE)^60^ generalized gradient approximation (GGA) functional was employed for the exchange and correlation interactions with ultrasoftpseudopotentials^61^. The cut-off Energy was chosen at 480 eV and a 3 × 3 × 1 Monkhorst-Pack (MP)^62^ k-points mesh while supercells of 108 atoms were adopted for the bulk system.The structure was optimized with the Broyden-Fletcher-Goldfarb-Shanno (BFGS) method. To consider the effects of electronlocalization, the DFT + U method was employed for spin-polarized calculations with on-site Coulomb repulsions of 8.2 eV for the 3d orbitals of Ti. Finally, for the DOS calculations, a 3 × 3 × 3 k-points mesh of was adopted while for the PDOS a 7 × 7 × 7. The efficacy of the present approach has been demonstrated in recent studies^10,11^. For the surface structures, a supercell consisting of 96 atoms was used, with an energy cut off of 480 eV and a MP k-point mesh of 2 × 2 × 1. Finally, for the density of states, we chose a 7 × 7 × 7 k-point mesh.
